# Mechanical positioning of multiple nuclei in muscle cells

**DOI:** 10.1371/journal.pcbi.1006208

**Published:** 2018-06-11

**Authors:** Angelika Manhart, Stefanie Windner, Mary Baylies, Alex Mogilner

**Affiliations:** 1 Courant Institute of Mathematical Sciences, New York University, New York, New York, United States of America; 2 Program in Developmental Biology, Sloan Kettering Institute, Memorial Sloan Kettering Cancer, New York, New York, United States of America; Rice University, UNITED STATES

## Abstract

Many types of large cells have multiple nuclei. In skeletal muscle fibers, the nuclei are distributed along the cell to maximize their internuclear distances. This myonuclear positioning is crucial for cell function. Although microtubules, microtubule associated proteins, and motors have been implicated, mechanisms responsible for myonuclear positioning remain unclear. We used a combination of rough interacting particle and detailed agent-based modeling to examine computationally the hypothesis that a force balance generated by microtubules positions the muscle nuclei. Rather than assuming the nature and identity of the forces, we simulated various types of forces between the pairs of nuclei and between the nuclei and cell boundary to position the myonuclei according to the laws of mechanics. We started with a large number of potential interacting particle models and computationally screened these models for their ability to fit biological data on nuclear positions in hundreds of *Drosophila* larval muscle cells. This reverse engineering approach resulted in a small number of feasible models, the one with the best fit suggests that the nuclei repel each other and the cell boundary with forces that decrease with distance. The model makes nontrivial predictions about the increased nuclear density near the cell poles, the zigzag patterns of the nuclear positions in wider cells, and about correlations between the cell width and elongated nuclear shapes, all of which we confirm by image analysis of the biological data. We support the predictions of the interacting particle model with simulations of an agent-based mechanical model. Taken together, our data suggest that microtubules growing from nuclear envelopes push on the neighboring nuclei and the cell boundaries, which is sufficient to establish the nearly-uniform nuclear spreading observed in muscle fibers.

## Author summary

How the cell organizes its interior is one of the fundamental biological questions, but the principles of organelles’ positioning remains largely unclear. In this study we use computational modeling and image analysis to elucidate mechanisms of positioning of multiple nuclei in muscle cells. We start with the general hypothesis, supported by published data, that a force balance generated by microtubule asters growing from the nuclei envelopes are responsible for pushing or pulling neighboring nuclei and cell boundaries, and that these forces position the nuclei. Instead of assuming what these forces are, we computationally screen all possible forces by comparing predictions of hundreds simple mechanical models to experimentally measured nuclear positions and shapes in hundreds of *Drosophila* muscle cells. This screening results in the model, according to which microtubules from one nucleus push away both neighboring nuclei and cell boundaries. We also perform detailed stochastic simulations of the only surviving model with individual growing, pushing and bending microtubules. This model predicts subtle features of nuclear patterns, all of which we confirm experimentally. Our study sheds light on general principles of organelle positioning.

## Introduction

One of the fundamental challenges of cell biology is to define principles of spatial organization of the cell [1], and, in particular, to unravel the mechanisms that control the position, size, and shape of organelles. The nucleus is the principal organelle and organizational center of eukaryotic cells. In textbooks, it is typically depicted in the middle of the cell; however, the nucleus’ actual position, (as in the apical/basal position in developing neuroepithelia [2]), depends on the cell’s migratory state, cell cycle stage, and differentiation status [3]. Proper nuclear position is vital for many cell functions, including spatially correct cell division and the direction of cell migration [3].

Multinucleation is one mechanism adopted by cells to generate and sustain large cell sizes. Muscle cells are one of the largest cell types, which are formed by fusion of mononucleated myoblasts and contain up to several tens (invertebrates) to several hundred (vertebrates) nuclei. Myonuclei are typically positioned at the cell’s periphery, and are distributed to maximize internuclear distance. However, in muscles undergoing repair, they are found towards the cell center, and in muscle diseases known as Centronuclear Myopathies, myonuclei are also found to be mispositioned [4, 5]. It has been argued [6], that correct positioning of myonuclei is not only an indicator, but also a cause of muscle diseases. A possible mechanism is provided by the *Myonuclear Domain Hypothesis* [7, 8], which suggests that each nucleus caters for a particular domain of the cell by making the gene products locally needed. Mispositioned nuclei would consequently not be able to guarantee the correct supply of products to their cytoplasmic domains, affecting muscle function.

In this work, we focus on nuclear positioning mechanisms in multinucleated muscle fibers. *Drosophila* is a good in vivo model system for investigating muscle development, growth, and homeostasis [9–11], due to the simplicity of its muscle pattern, the ease of genetic manipulation, and the homology of relevant genes and processes to mammalian muscle. Nuclei in newly fused *Drosophila* embryonic muscle cells undergo an orchestrated series of movements, best described in lateral transverse muscles: after fusion of the myoblasts, the resulting muscle cell is thought to disassemble its centrosomes and redistribute *γ*-tubulin around each nuclear envelope. The myonuclei initially form a cluster close to the cell center. The cluster splits into two subclusters that then migrate towards the opposing cell poles. Subsequently both clusters break apart, and the nuclei spread out evenly along the cell long axis [12, 13]. As the nuclei spread in the muscle cell, sarcomeres, the fundamental contractile units in muscle, form into myofibrils within each cell, and, at the end of embryogenesis, the nuclei become positioned along the long axis of the cell at its periphery, thereby maximizing internuclear distance. During the subsequent larval stages of development, the muscle cells grow 20-40 fold over the course of 5 days without the addition of new myonuclei [14]. Nevertheless, the myonuclei remain appropriately positioned along the cell, although the mechanisms that are responsible for this are not clear.

While the actomyosin network may be involved in nuclear positioning [15], microtubules (MTs), MAPs (MT Associated Proteins), and MT-based motors, such as kinesin and dynein, have been shown to play a major role [12, 16–18]. As examples, in embryos in which MTs are severed in the muscle cell, the central cluster does not split; in many motor mutants, nuclear spreading in the muscle cell is perturbed [19]. However, the precise mechanisms controlling myonuclear positioning remain poorly understood.

Modeling has proven to be very useful in complementing cell biological methods in problems of positioning with, for example, the mitotic spindle [20, 21]. Mathematical modeling focused on multinucleated cells and nuclear positioning is in its infancy. Simple conceptual models of nuclei repelling each other were used in [22] and [23] to show that such models can explain regular distribution of nuclei in muscle cells and in the *Drosophila* blastoderm syncytium. Detailed mechanical simulations were done in [24] to understand multiple nuclear movements in multinucleate fungus *Ashbya gossypii*.

Here, we use computational modeling to understand the mechanisms regulating nuclear positioning in *Drosophila* larva muscle cells. We hypothesize that nuclear positioning is a result of a MT-motor based force balance. Rather than assuming the nature of this force balance, we screened multiple computer-generated forces by comparing the spatial nuclear patterns that they predict to quantitative microscopy data from biological specimens. We then simulated a detailed agent-based model to confirm the predictions of the screen. One model explains all biological data, including many subtle patterns of multi-nuclear positioning. Based on this model we propose that myonuclei are positioned by establishing a force balance via MT-mediated repulsion.

## Results

### Experimental data

**Fig 1:**
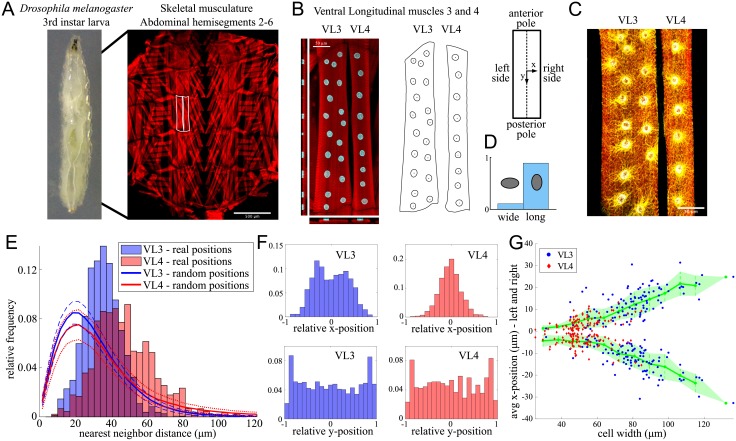
Positioning of the myonuclei in Ventral Longitudinal (VL) muscles 3 and 4. A: Left: *Drosophila* 3^rd^ instar larva (anterior up), right: Dissected larva revealing the somatic musculature, labeled with phalloidin (red) to reveal actin structures (sarcomeres) in muscles. B: Left: Representative VL3 and VL4 muscles (phalloidin, red) and nuclei (*α*-Lamin, cyan), right: Measured cell and nuclear outlines and nuclear centroids. C: VL3 and VL4 muscles (phalloidin, red) showing organization of microtubules (*α*-tubulin, yellow) around myonuclei (DNA; Hoechst, gray). D: Frequency of wide vs long nuclei defined using the angle of the major axis of a fitted ellipse. E: Histogram of nearest neighbor distances (NND) in VL3 and VL4 muscles. Filled bars show the experimental values, blue for VL3, red for VL4 and dark-pink where they overlap; the red and blue curves show the computer-generated NND histogram for randomly positioned nuclei (1000 realizations for all 200 cells, thick-solid lines are the means, thin-dashed and thin-dotted lines the mean ± standard deviation for VL3 and VL4 respectively). F: Histograms of the nuclear positions along the *x*- and *y*-axes for both cell types. The half-length and half-width of the cells were normalized to 1. G: Average nuclear *x*-positions relative to the center for each cell as functions of the cell width. Shown are the averages (green lines) ± standard deviation (green shading) as well as the actual measurements for all VL3 (blue) and VL4 (red) cells.

We have focused on data obtained from Ventral Longitudinal (VL) muscles VL3 and VL4 of *Drosophila* 3^rd^ instar larvae (Fig 1A–1C). The 200 analyzed muscle cells (103 VL3 and 97 VL4 cells) contained between 6 and 23 nuclei (VL3: 15.3 ± 2.9, VL4: 9.3 ± 1.6). Representative confocal microscopy images of fixed samples with fluorescently labeled muscle cells (actin), myonuclei (lamin, Hoechst) and microtubules (alpha-tubulin) are shown in Fig 1A–1C. We used cells from three control genetic backgrounds (*w*^1118^, *Dmef2-GAL4;UAS-2xEGFP, Dmef2-GAL4;UAS-GFP RNAi*). VL muscles are flat rectangular cells with nuclei located on one cell side. We employed the following terminology: cell length is the dimension along the long axis of the cell (*y*-direction), cell width is the dimension along the short axis of the cellular rectangle (*x*-direction) (Fig 1B). We referred to the edge of the *z*-projection of the cell as the cell boundary, and distinguished between cell sides (long segments of the rectangular shape) and cell poles (short segments of the rectangle). We defined the subcellular localization of nuclei and measured several geometric parameters relevant to nuclear positioning, including nuclear numbers, shapes, nearest neighbor distances, and distances to the cell boundary, and used statistical tools to analyze the data.

The following features of VL muscle cells and nuclei informed our modeling: 1) Both cell types share a similar length (VL3: 499.2 ± 57.7*μm*, VL4: 491.8 ± 57.1*μm*), but VL4 cells’ width is significantly smaller (VL3: 80.4 ± 17.5*μm*, VL4: 51.7 ± 11.3*μm*). 2) Nuclei are not randomly positioned. We simulated random nuclear positioning by generating random and independently uniformly distributed *x*- and *y*-coordinates of the nuclear centers in rectangular domains. Analyzing the experimentally measured nearest-neighbor distances between the nuclei showed that the arrangement is not random (Fig 1E); rather, there is a characteristic distance between the neighboring nuclei. 3) Along the long axis of the cell, nuclei are relatively evenly spread, with a slight increase of the nuclear density near the cell poles (Fig 1F, lower row). 4) Along the short axis of the cell, nuclei tend to form a single file (SF) near the cell center in narrower VL4 cells, whereas in wider VL3 cells, nuclei are typically found in a double file (DF) arrangement (Fig 1F, upper row). In fact, the average nuclear *x*-position within each cell is a function of the cell width (Fig 1G): the nuclear *x*-position increases with increasing cell width, irrespective of the cell type. Thus, nuclei in a wide VL4 cell have the same position as those of a VL3 cell of equal width. 5) VL3 and VL4 nuclei have the shape of flat ellipsoidal discs with their long axis oriented along the cell’s long axis (Fig 1B and 1D). 6) Microtubules (MTs) anchored at the nuclear envelopes form asters around each nuceus (Fig 1C). When MT organization is disrupted, nuclei have been shown to be mispositioned [25], suggesting that MTs exert forces on the nuclei, and that the resulting force balance is the key to the nuclear positioning.

### Force-balance and force-screening

One general approach to modeling movements of cell organelles generated by MT-motor forces is “interacting particle modeling”. After assuming or calculating a mean MT-motor force between material objects in the cell as a function of distance between the objects, one can solve equations of motion for the interacting objects via position-dependent force laws. This leads to solving a system of ordinary differential equations (ODEs), the number of which is equal to the number of the objects [22, 23, 26, 27]. Such models can be simulated rapidly such that many different internuclear force types can be screened.

Another approach is to avoid assumptions and approximations and to formulate a “detailed agent-based model”, which involves solving numerically equations of elasticity theory for each MT, together with equations of motions for motors and objects to which MTs and/or motors are anchored [24]. This leads to solving a large system of partial differential equations (PDEs), or a gigantic ODE system, which is much more difficult and computationally expensive than the first approach. While this approach provides more detailed predictions, many motors are involved in the force balance [19], and it is not clear which combination of the motors generates the force in this context. Further, mechanical characteristics of most of these motors are unknown. In principle, one could use all possible motor combinations [28], but the long computation times and high dimensionality of the model parameter space makes parameter scans of the detailed stochastic model impossible [29].

In this work, we systematically combine the two approaches to suggest mechanisms of nuclear positioning. We begin by screening various forces using interacting particle modeling and determine which model can be responsible for not only qualitative features of the observed spatial patterns, but also explain quantitatively all the subtle geometry of the nuclear positioning. Such an unbiased and systematic computational screen was needed for a few reasons. First, there are multiple simple and intuitive models that predict roughly uniform nuclear distribution, and choosing between them by a traditional thinking process is vulnerable to psychological biases. Second, spatial patterns generated by simple forces may be highly complex, counterintuitive, and non-robust [30]. After this screen, we used learned force characteristics to inform a detailed agent-based stochastic model with explicit simulations of individual MTs to confirm the lessons from the screening.

#### The interacting particle model

We make the following assumptions (A1-5) for the interacting particle models. A1: The cell can be described as a 2D rectangle. A2: The velocity of a nucleus is proportional to the total force acting on this nucleus. This is typical in a viscous friction-dominated environment in cells characterized by low Reynolds numbers [20, 21, 23, 24]. A3: Nuclei interact with each other via pairwise interactions; further, independent interactions exist between each nucleus and the cell boundary. A4: All interactions are deterministic, isotropic and result in distance-dependent forces discussed below. A5: The forces are additive: movement of the *i*-th nucleus at each time moment is determined by the sum of the forces acting on it from all other nuclei as well as from the cell sides and cell poles.

**Fig 2:**
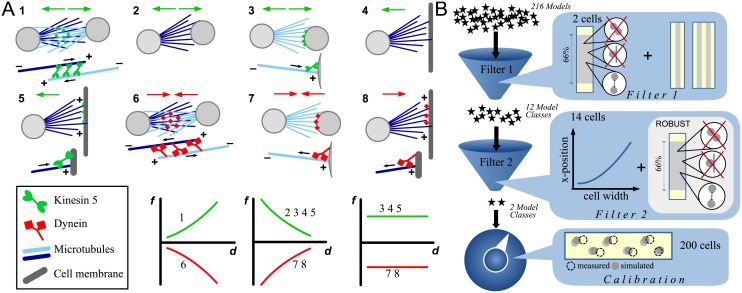
Force types and force screen. A: Possible molecular mechanisms of nuclear interaction via MT generated forces (1-8; see key bottom left, gray circles represent nuclei). Different forces *f* (positive for repulsive, negative for attractive) as a function of distance *d* are depicted in the lower row. 1: Kinesin 5 acting between overlapping antiparallel MTs from neighboring nuclei generates a repulsive force. Greater distances between the nuclei lead to a larger MT overlap (assuming long MTs), hence the force is increasing with distance. 2: MTs growing from one nucleus push on the neighboring nucleus, resulting in a repulsive force that drops with distance, since MT ends’ density decreases with the distance. 3: Kinesin motors are localized at the nuclear membrane and push away MT plus ends from neighboring nuclei. Depending upon whether the number of MTs or the number of kinesins are limiting, the resulting repulsive force can be decreasing with distance or be distance independent. 4 and 5: As 2 and 3, but the nucleus interacts with cell boundary or with motors on the cell cortex at the cell boundary. 6 and 7: Analogous to 1 and 3, but with the motors being dyneins (or kinesin 14), rather than kinesins, resulting in an attractive force. 8: Analogous to 7, but MTs from the nucleus interact with dyneins on the cell cortex at the cell boundary. B: Structure of the force screen: Two filtering steps are followed by a calibration step. We started with all 216 possible models and made a rough scan of the each model’s parameter space using a representative thin (VL4) cell and a representative wide (VL3) cell. Filter 1: The models are filtered by their ability to produce an evenly spread single file (SF) of nuclei in the thin cell, and double file (DF) in the wide cell. We found that the vast majority of the models fail this step and can be discarded; only 12 potential model classes remained. Filter 2: We applied each of the 12 model classes to 14 imaged cells of representative width, height and number of nuclei and examined whether the models can account for two characteristics of the biological data: 1) average nuclear *x*-position increases with cell width, 2) the model behavior is robust with respect to parameter changes. After this second filter, only two model classes remained. In the final step (“calibration”), we fixed the pole forces and used the data on the nuclear positions from all 200 imaged cells to determine the best parameters for the two models.

Assumptions 1 is well justified by the biological data. With regards to A2, we note that MT entanglement could cause an effective additional internuclear friction [23], in which the speed of a nucleus is influenced by the speeds of neighboring nuclei. We tested such a scenario (see S1 Text) and found that while myonuclear movement is affected by this additional friction, there seems to be very little effect on the final nuclear positions. Since this study is concerned with equilibrium positions only, we decided to omit the internuclear friction; however, it might be an important consideration for future work dealing with transient dynamics. We discuss the validity of A3-5 in Sec Comparison to agent-based, stochastic simulations; here we briefly comment on origins of these three assumptions. Examples of molecular mechanisms shown in Fig 2A invoke forces generated by stochastic processes of MT growth and motor action. However, as the MT and motor numbers per nucleus are on the order of hundreds, if not thousands, and the MT and motor kinetics are much faster than nuclear movements (motors bind/unbind on a second time scale; MT dynamic instability occurs on tens of second time scale; nuclear movements take tens of minutes), the resulting net forces per nucleus are likely to be almost deterministic [28]. Assuming radially symmetric MT and motor distributions on nuclear envelopes, the forces are approximately isotropic. The pairwise and additive character of the forces is based on the scenario in which one set of MTs from one nucleus interacts with the second nucleus, while another MT set from the first nucleus interacts with the third nucleus or the cell boundary; in this case, the force on the first nucleus is equal to the sum of two independent forces from the second and third nuclei respectively. However, flexible interacting MTs and possibility of the second nucleus being between the first and third one complicate this picture. Only a full stochastic model can address the full complexity.

The model’s dynamic variables are the positions of *N* nuclei in a rectangular cell with width 2*b* and length 2*l*; the position of the *i*-th nucleus at time *t* is a 2D variable *X*_*i*_(*t*) ∈ [−*b*, *b*] × [−*l*, *l*]. We denote by *f* the (scalar) forces between pairs of nuclei, and by *g*_S_ and *g*_P_ the (scalar) forces between a nucleus and the cell sides or a nucleus and the cell poles, respectively. Since we are only interested in equilibrium nuclear positions in this study, we normalized the effective drag coefficient characterizing the nucleus to unity. Thus, the velocity of the *i*-th nucleus is equal to the sum of all forces acting on it:

$$\begin{array}{ll} \frac{\text{d}X_{i}}{\text{d}t}= & \sum_{\begin{array}{c} j=1\\ j\neq i \end{array}}^{N}f(\left\|X_{i}-X_{j}\right\|)\frac{X_{i}-X_{j}}{\left\|X_{i}-X_{j}\right\|}+\underset{k\in \left\{L,R\right\}}{\sum }g_{\text{S}}(\left\|X_{i}-w_{i}^{k}\right\|)\frac{X_{i}-w_{i}^{k}}{\left\|X_{i}-w_{i}^{k}\right\|}\\ & +\sum_{k\in \left\{U,D\right\}}g_{\text{P}}(\left\|X_{i}-w_{i}^{k}\right\|)\frac{X_{i}-w_{i}^{k}}{\left\|X_{i}-w_{i}^{k}\right\|}, \end{array}$$ (1)

$$\begin{array}{c} X_{i}\left(0\right)=X_{i}^{0},\;i=1,..,N \end{array}$$

Here $w_{i}^{k}$ is the normal projection of the 2D position of the *i*-th nucleus on the corresponding cell boundary, the letters *U*, *D*, *L*, *R* denote the cell boundary up, down, left and right of the nucleus respectively. ‖.‖ denotes the length of a 2D vector. The addition of a size exclusion term is described in the Methods.

#### Forces

To complete the system of Eq (1), we specified the distance dependence of forces *f*, *g*_S_ and *g*_P_. Fig 2A depicts possible molecular mechanisms motivating different force distance dependencies; the figure legend explains the molecular details. These examples produced either purely repulsive or attractive forces, which can be either increasing or decreasing with distance or are distance-independent. Additionally, it is reasonable to assume that in some cases (when MTs grow only to a certain length), forces may have only a finite reach. To represent the forces mathematically, we used the expressions:

$$f\left(d\right)=\sigma_{N}{\left(\frac{d}{d_{\text{ref}}}\right)}^{\alpha_{N}}H\left(c_{N}-d\right)$$

$$\begin{array}{cc} & g_{S}\left(d\right)=\sigma_{S}M_{S}{\left(\frac{d}{d_{\text{ref}}}\right)}^{\alpha_{S}}H\left(c_{S}-d\right),\;g_{P}\left(d\right)=\sigma_{P}M_{P}{\left(\frac{d}{d_{\text{ref}}}\right)}^{\alpha_{P}}H\left(c_{P}-d\right). \end{array}$$

Here *d* is the variable distance, and parameter *d*_ref_ = 40*μm* is the typical distance between neighboring nuclei used to scale the variable distance. The sign *σ*_*j*_ = ±1 describes whether the force is repulsive *σ*_*j*_ = +1 or attractive *σ*_*j*_ = −1. To describe the finite reach of forces, we used cut-off values *c*_*N*,*S*,*P*_ > 0; *H*(.) denotes the Heaviside function (*H*(.) = 1, 0 for positive, negative values of the argument, respectively). The constants *M*_*S*,*P*_ > 0 define the relative magnitude of the side and pole forces compared to the internuclear forces. The exponents *α*_*N*,*S*,*P*_ characterize the behavior of the force with distance: decreasing for *α*_*j*_ < 0, constant for *α*_*j*_ = 0 and increasing for *α*_*j*_ > 0. In the computational screens, we restricted the values of the exponents to *α*_*j*_ ∈ {−1, 0, 1}.

Of course, the effective forces of interactions mediated by complex MT behavior and multiple motor types may be more complicated: these may be repulsive at some distances and attractive at others, have distance dependencies other than the power laws [26, 28], and even have non-monotonic distance dependence [31]. To address these possibilities, we did numerical experiments with force expressions, including exponents other than those used in the screen, and with functions other than power laws, including exponential functions and others. We found that all qualitative results of the screen remained valid after such variations. We also experimented with forces that are repulsive at some distances and attractive at others (see S1 Text), and found that as long as the long-ranged forces are relatively small, they lead to results similar to those predicted by purely repulsive or attractive forces. As for more complex possibilities of non-monotonic distance dependence, these are beyond the capabilities of our approach. In the future, if data suggest such possibilities, it will have to be considered then.

We adopted the following terminology: a *model* is defined by a unique combination of parameters *σ*_*N*_, *σ*_*S*_, *σ*_*P*_, *α*_*N*_, *α*_*S*_, *α*_*P*_. In other words, each combination of three repulsive or attractive forces, each characterized by being either decreasing or increasing with distance or distance independent, is a model. This gives 2^3^ × 3^3^ = 216 different models. In addition, each model is characterized by two relative force amplitude parameters *M*_*S*,*P*_ and by three force range parameters *c*_*N*,*S*,*P*_. We also found that the exact form of the pole force has little effect on the nuclear distribution, as far as the pole force has a range much smaller than the cell length. Therefore, we also used the term *model class*, which defines a number of models that are characterized by a combination of parameters *σ*_*N*_, *σ*_*S*_, *α*_*N*_, *α*_*S*_. There are 2^2^ × 3^2^ = 36 model classes.

#### Force screen structure

We found that the screen works best if executed in two filtering steps, which reduced the number for initial models from 216 to 12 (Filter 1) and then further to two (Filter 2). This is followed by a calibration step. The steps of the force screen are summarized in Fig 2B and detailed below. Analyzing the final two models using statistical and analytical methods, we then made a number of predictions that we compared to the features of the experimental data that we did not consider in the filtering rounds (Sec Models’ predictions). This allowed us to select and characterize the ultimate model.

#### First filtering step

**Fig 3:**
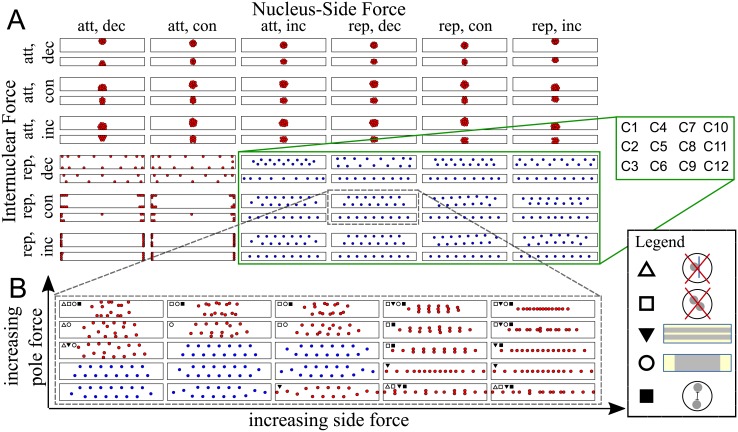
Sample gallery of the spatial nuclear patterns produced in Filter 1. A: Equilibrium positions for the 36 model classes (examples). Combinations of forces (att: attractive, rep: repulsive, dec: decreasing, inc: increasing, con: constant). We show results of varying the character of internuclear and nuclear-cell side forces. For each force combination, we show predicted patterns in a narrow (VL4) and wide (VL3) cell (black rectangles). Nuclei at their equilibrium positions are shown as blue and red discs for valid and non-valid patterns respectively. Outside of the green box, the models have not passed the first filtering step for any parameter values. The examples are shown for parameter values *c*_*N*_ = *c*_*S*_ = *c*_*P*_ = ∞, *σ*_*P*_ = 1, *α*_*P*_ = −1 and *M*_*s*_ = *M*_*p*_ = 1. Green box: model classes that passed the first filtering step for some parameter combination (positions shown represent valid patterns). For one particular combination (grey, dashed box), further details of scanning the parameter space are shown in B. B: Equilibrium positions for the (wide) VL3 cell for the force combination marked in gray in A, for all combinations of force ratios (*M*_*s*_, *M*_*p*_) for distance independent repulsive internuclear forces (with range *c*_*N*_ = 50), and for repulsive side and pole forces falling with distance (with an infinite range). Blue nuclei indicate valid patterns. For all non-valid patterns (red nuclei), symbols indicate the type of violation: △—nuclei stick to the edges, ◻—nuclei are too close together, ▼ nuclei don’t form two files, ○—nuclei do not stretch 2/3 of the cell’s length, ◼—mean nearest neighbor distance is less than 30*μm*. For parameters see Table 1, for details see Methods.

To screen for models capable of reproducing the observed patterning, we simulated each of 216 models multiple times, scanning roughly each model’s parameter space. Tab 1 lists all parameters used. Each model was tested with typical VL3 (wide) and VL4 (thin) cell geometries, starting with both randomly placed and equally spaced nuclei. Counting all parameter combinations, the total number of simulations was: 216 models × 4^3^ force ranges × 5^2^ force ratios × 2 geometries × 2 initial conditions ≈ 1.4 ⋅ 10^6^. Each simulation was run until an equilibrium was reached and the resulting spatial patterns were evaluated using the following criteria (Filter 1 in Fig 2B, legend in Fig 3, for details see Methods):

**Table 1:** Parameters used for the two filtering steps and the calibration step.

Parameter	Values used	Comment
**Filter 1**		
*σ*_*j*_, *j* = *N*, *S*, *P*	−1, 1	attractive or repulsive
*α*_*j*_, *j* = *N*, *S*, *P*	−1, 0, 1	falling, constant, increasing with distance
*M*_*j*_, *j* = *S*, *P*	5 values between 0.1 and 10	force ratio
*c*_*j*_, *j* = *N*, *S*, *P*	25, 50, 110, ∞	*c*_*j*_ = ∞ means forces reach across entire cell
2*l*	500	fixed cell height
(2*b*, *N*)	(80, 15), (50, 9)	corresponds to VL3 and VL4 cell
$X_{i}^{0}$, *i* = 1..*N*	random, equally spaced along *x* = 0	initial condition
**Filter 2**		
(*σ*_*P*_, *α*_*P*_, *c*_*P*_, *M*_*P*_),	(1, −1, 25, 200)	fixed pole forces
(*σ*_*N*_, *α*_*N*_)	(1, −1), (1, 0), (1, 1)	correspond to C1-12
(*σ*_*S*_, *α*_*S*_)	(−1, 1), (1, −1), (1, 0), (1, 1)	correspond to C1-12
*M*_*S*_	7 values between 0.1 and 10	force ratio
*c*_*j*_, *j* = *N*, *S*	11 values between 20 and 500	
$X_{i}^{0}$, *i* = 1..*N*	random	initial condition
2*l*	500	fixed cell height
*N*	7, 8, 9, 10, 11, 12, 13, 14, 15, 16, 17, 18, 20, 23	number of nuclei
*b*	42, 47, 51, 55, 57, 70, 71, 73, 83, 85, 88, 91, 113, 117	cell widths
**Calibration**		
(*σ*_*P*_, *α*_*P*_, *c*_*P*_)	(1, −1, ∞)	pole forces
(*σ*_*N*_, *α*_*N*_, *c*_*N*_)	(1, −1, ∞)	correspond to M1, M2
(*σ*_*S*_, *α*_*S*_, *c*_*S*_)	(−1, 1, ∞), (1, −1, ∞)	correspond to M1, M2
*M*_*j*_, *j* = *S*, *P*	13 values between 0.1 and 10	force ratio
(2*l*, 2*b*, *N*)	200 triples	corresponding to biological measurements
$X_{i}^{0}$, *i* = 1..*N*	as measured	initial condition
**Calibration Result**		
(*σ*_*P*_, *α*_*P*_, *c*_*P*_, *M*_*P*_),	(1, −1, ∞, 3.2)	pole forces
(*σ*_*N*_, *α*_*N*_, *c*_*N*_)	(1, −1, ∞)	M1, M2
(*σ*_*S*_, *α*_*S*_, *c*_*S*_, *M*_*S*_)	(−1, 1, ∞, 2.2), (1, −1, ∞, 2.2)	M1, M2

To pass Filter 1, nuclei 1) were not allowed to stick to each other, 2) were not allowed to stick to the cell sides or poles, 3) were required to spread along the long axis of the cell, in the sense that the distance from the maximal to the minimal *y*-coordinate of the nuclei had to be at least 2/3 of the cell length. 4) the mean NND (Nearest Neighbor Distance) had to be above 30*μm* and 45*μm* for the VL3 and VL4 cell respectively—this avoids counting random positioning as correct. 5) For the VL4 cell, the histogram of the nuclear *x*-coordinates had to have a single peak in the middle of the cell; for VL3 cells, we required the histogram to be double peaked. Fig 3 shows the result of the first filtering step. We found that 24 of the 36 modeling classes did not satisfy all five criteria imposed by the biological data for any values of the model parameters (Fig 3A). Twelve model classes passed Filter 1 successfully, in the sense that we found parameter values for these model classes for which all five criteria imposed by the data were satisfied (examples shown in in Fig 3B).

These 12 classes, C1-12, were characterized by the internuclear and nucleus-cell side forces; pole forces played a lesser role. We found that in these model classes, the nuclei repel each other; interestingly, this repulsion can be distance independent or even increasing with distance, as long as the internuclear force range is around 50*μm*. For the nuclear-cell side forces, a variety of forces are possible: attractive forces increasing with distance (spring-like forces) that reach across the width of the cell, as well as all types of repulsive forces. For the distance independent repulsion or repulsion increasing with distance, the side forces need to have a very specific reach of about half the width of a typical VL4 cell. If the reach is above or below that, nuclei in VL4 cells do not align along the center of the cell.

#### Second filtering step

**Fig 4:**
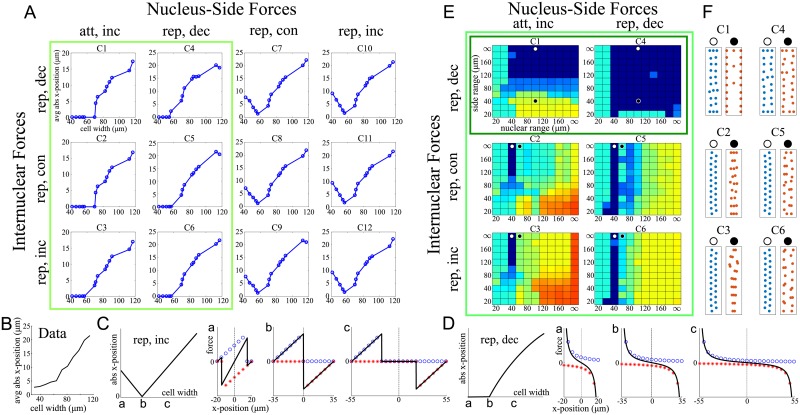
Elimination steps in Filter 2. A: Average absolute *x*-position of the nuclei as a function of the cell width, predicted by 12 model classes that passed Filter 1. The calculations were made for the force ranges that ensured that the criteria of Filter 1 (not using the SF/DF criteria, see Methods for details) were satisfied and for the force magnitude that minimized the error of the fit of the calculations to the biological data. The light green rectangle marks those model classes for which the average absolute *x*-position increases with cell width as observed in the data (see B). B: Measured dependence of the average absolute *x*-position on the cell width. C: Effect of repulsive, increasing with distance side forces with a finite range for three different cell widths (a,b,c). Blue circles and red stars show the individual contributions from the left and right side respectively, the black lines in a,b,c show the resulting force felt by a nucleus at position *x* (positive/negative forces cause movement to the right/left). D: As C, but for repulsive side forces, decreasing with distance. E: For each model class and each combination of force ranges, the color represents how well the four criteria depicted in Fig 2B, Filter 2, are fulfilled. Dark Blue = all criteria fulfilled for all 14 cells, Red = all are violated. The dark green rectangle marks those model classes which were examined further. White and Black circles mark examples shown in F. F: Examples for sensitivity on the force range for one of the 14 cells. Shown are one example with valid positioning pattern (white circle) and one with invalid patterning (black circle), which the exception of C4, where both patterns are valid.

To further reduce the number of models and test for biological relevance, we used the positioning results of a 14-cell screen. Specifically, we used the dimensions and nuclear numbers measured in 14 representative cells (see the list and parameters in Table 1, applied the 12 remaining model classes to each of these 14 cells, and evaluated the nuclear positions according to two criteria: 1) Does the average absolute nuclear *x*-position increase with cell width? (Fig 4A) 2) How robust is the model with respect to changes in parameter values (Fig 4E). Focusing on the interplay between internuclear and side forces, we fixed the pole forces to short-ranged repulsive forces (compare Table 1). To apply the first criteria, we used the sets of parameters (force magnitude and range) for each of the modeling classes that provided a minimal error in predicting the computed average absolute nuclear *x*-position of the experimental data (Fig 4B). The results shown in Fig 4A led to the exclusion of model classes C7-12, since these models predict that the average absolute nuclear *x*-position initially decreases with cell width, in contrast to the biological data. An intuitive explanation for this behavior is depicted in Fig 4C for the example of repulsive, increasing with distance side forces, with a finite range: For very thin cells the side forces from the right and left overlap, creating a region in the middle of the cell, where the nucleus feels an effective decentering force. Only a very specific cell width will promote centering (explaining the dip in the width vs *x*-position plot). Finally, for wide cells, nuclei near the cell center will feel no side forces, however, since they will be pushed by other nuclei, this will again lead to an *x*-position away from the center. In comparison, repulsive side forces, decreasing with distance (Fig 4D) will always promote centering, however as cells get wider, these forces reduce and eventually neighboring nuclei succeed in pushing the nucleus out of the center. We will examine this balance further in Sec Models’ predictions. Out of the remaining 6 model classes, we further excluded all models with repulsive, increasing or constant internuclear forces (C2,3,5,6) from the robustness point of view, since even small deviations from the 40*μm*-range of the force caused defects in the positioning patterns (Fig 4E): Greater force range led to clustering, while smaller range caused a lack of the nuclear spreading along the *y*-axis. Fig 4F shows examples of such positioning defects.

#### Two model classes result from the screens

The two filtering steps resulted in just two model classes that can predict robust nuclear spreading along the cell long axis and correct behavior of *x*-positions with respect to cell width:

*Model Class 1:* The internuclear forces are repulsive and decrease with internuclear distance. The forces between the nuclei and cell sides are attractive, increasing with distance and reaching across the entire cell width. This model class is characterized by parameters *σ*_*N*_ = 1, *α*_*N*_ < 0, *σ*_*S*_ = −1, *α*_*S*_ > 0.

*Model Class 2:* The internuclear forces are repulsive and decrease with internuclear distance, as in the first model. The forces between the nuclei and cell sides, however, are repulsive, decreasing with distance. This model class is characterized by parameters *σ*_*N*_ = 1, *α*_*N*_ < 0, *σ*_*S*_ = 1, *α*_*S*_ < 0.

#### Calibration

To calibrate the two model classes (i.e. find parameters of the force magnitudes *M*_*S*_ and *M*_*P*_), we applied the models to all 200 imaged cells, using as initial conditions, the measured nuclear positions from the biological data, and as an error measure the mean absolute deviations of the model-predicted equilibrium positions from the measured nuclear positions. We also assumed that the cell poles repelled the nuclei with forces decreasing with distance. The resulting model parameters are shown in Table 1. This calibration procedure resulted in two single models (not classes) with precisely determined parameters, which can now be tested by further comparison with the data. In the following, we will refer to them as Model 1 (M1) and Model 2 (M2).

### Models’ predictions

#### Nuclear density is higher near the poles

**Fig 5:**
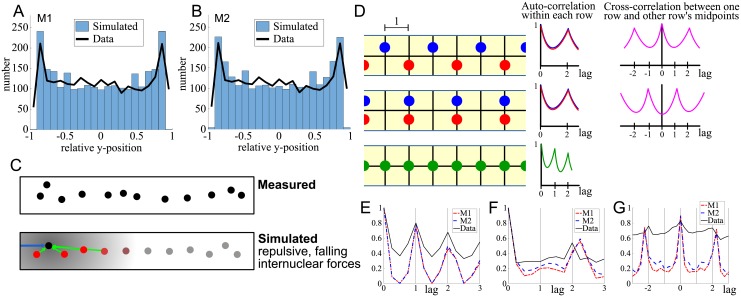
Pattern along the *x*- and *y*-axis. A and B: Comparison between the measured and simulated histogram of relative *y*-positions for M1 and M2 using the calibrated parameter values (Table 1). C: Example cell with the positions of the nuclei as measured (upper row) and determined by M1 (lower row). In the lower row, the forces felt by a nucleus near the pole (black) is shown. Blue arrow represents the pole force on the black nucleus; green arrows show internuclear forces; side forces are not depicted. The strength of internuclear forces is represented by varying shades of red. Gray shading represents the reach of the internuclear force. D: Assuming a perfect pattern of zig-zags (top row), pairwise files (middle row) or of equally spaced single files (bottom row), the resulting auto-, or cross-correlation is depicted on the right. E: The auto-correlation function predicted and measured for SF cells. F: The predicted and measured auto-correlation function for each row in DF cells. G: The predicted and measured cross-correlation between the left row and the middle points of the right row in DF cells. For details see Methods.

A notable feature of the experimental data is that along the long cell axis (*y*-direction) relatively more nuclei are found near the cell poles (Fig 1F lower row). Both models 1 and 2 accurately and quantitatively reproduce the nuclear density increase near the poles (Fig 5A and 5B). The internuclear forces have to be long-ranged to quantitatively fit the nuclear patterns in the imaged cells. A simple explanation for this phenomenon is that the nuclei near the cell poles feel more repulsion from the cell interior than from the cell poles, compared to nuclei in the interior where this repulsion force is more symmetric (Fig 5C). This repulsion pushes the nuclei at the edge into the cell pole, effectively crowding the nuclei to the cell ends.

#### Zig-zag patterns in the double file of the nuclei in wide cells

The experimental data demonstrates that along the cell’s short axis (*x*-direction) two patterns emerge: nuclei are arranged either in one line in the middle of the cell, or in two rows, one to the left, another to the right from the long axis of the cell (Fig 1F upper row). We refer to these patterns as *single file*, SF, and *double file*, DF, respectively. We used histograms of the nuclear *x*-position to classify each cell as SF, DF or neither (compare Methods).

Qualitatively, it was easy to understand why the DF pattern emerges. In closely positioned nuclei in the SF row, the repulsion between the neighbors tended to increase the internuclear distances, effectively squeezing the nuclei from the center of the cell toward the sides. A zig-zag pattern emerges as nuclei alternatingly move to the left and right, to maximize the distance to their neighbors. Numerical simulations confirmed that both models 1 and 2 make the following similar nontrivial predictions: nuclei in SF cells, as well as nuclei within each row in DF cells, are equally spaced in the sense that the autocorrelation function for the nuclear distribution in the single row has equidistant peaks (Fig 5E and 5F). Furthermore, the two nuclear rows in DF cells form the zig-zag pattern (Fig 5D): the *y*-coordinates of the nuclei in one row are shifted in relation to the *y*-coordinates of the nuclei in the other row. This was revealed by the cross-correlation between simulated nuclear positions in one row and the midpoints of nuclei in the other row in DF cells (Fig 5G). Repeating this analysis with the measured data, we found that M1 and M2 correctly predict both the SF in thin cells and the zig-zag pattern of DF cells (Fig 5E–5G).

#### Transition from single to double file in the nuclear pattern

An intuitive explanation for the transition between the SF and DF patterns suggested a way to derive approximate analytical expressions for the *x*-coordinates of the nuclear positions as functions of the cell width and of the nuclear spacing along the *y*-direction. The key was to make the following simplifying assumptions: 1) The nuclei are evenly spaced in the *y*-direction (spacing of λ = 2*l*/(*N* + 1), where 2*l* is the cell length, and *N* is the number of the nuclei). Essentially, we view the cell as an infinitely long strip. 2) Only the nuclear interactions with the cell sides and with their immediate neighbors (neglecting pole forces) are considered. 3) We consider the zig-zag configuration: If the *x*-position of a nucleus is *x*_*i*_, then its neighbors above and below have *x*-positions −*x*_*i*_.

**Fig 6:**
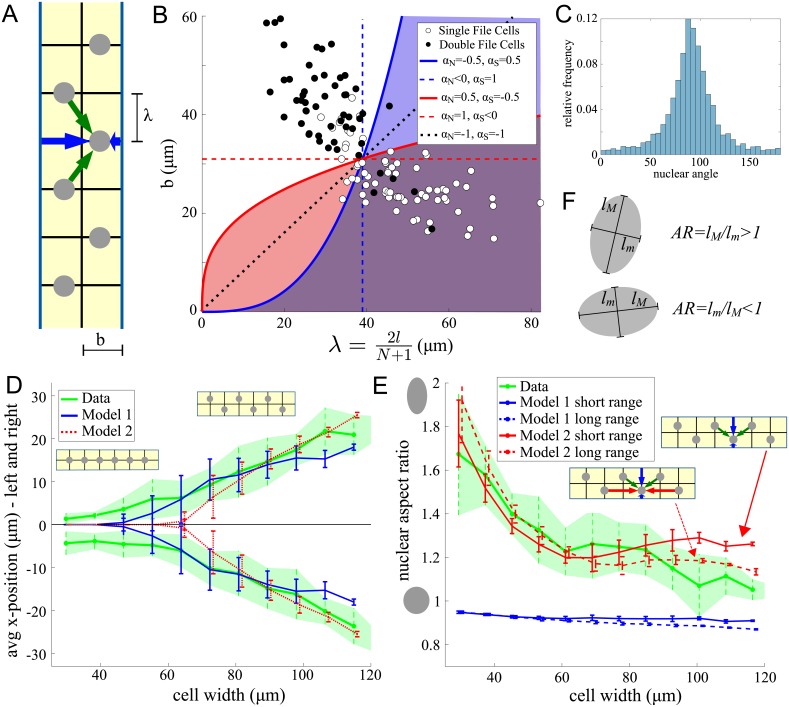
Nuclear file formation and nuclear shapes. A: The simplified *x*-position model. Green arrows show the forces on a given nucleus from the neighboring nuclei; blue arrows show forces between the given nucleus and cell sides. B: The geometry of the cell can be described by the *y*-spacing λ (determined by the cell height 2*l* and number of nuclei *N*) and the half width *b*. Each circle depicts one cell classified as single file (white) or double file (black) in (λ, *b*) space. Lines give the bifurcation values as predicted by Eq (3) for various values of *α*_*N*_ and *α*_*S*_, dashed lines show the limiting cases *α*_*N*,*S*_ = 1. M1 corresponds to dashed blue, M2 to dotted black. The constant given by the right hand side of Eq (3) was determined to match the behavior of the data. Shaded regions mark the predicted single file regime. C: Histogram of nuclear angles D: The measured and analytically calculated *x*-positions of the nuclei for Models 1 and 2 using Eq (4). The constants *M*_*S*_ were determined by minimizing the error of predicted *x*-positions compared to the data. This minimization yielded parameters *M*_*S*_ = 1.93, 1.88 for Models 1, 2, respectively. E: For each cell, the computed nuclear aspect ratio as given by Eq (5) was computed by finding the value of parameter *κ* by minimizing the error of the predicted aspect ratio compared to the data. The minimization yielded *κ* ≈ 35 in both models. For each case the average ± standard deviation is plotted as a function of cell width. F: Definition of the oriented aspect ratio of the nucleus.

Fig 6A illustrates these assumptions which are well supported by both the numerical simulations and the correlation analysis shown in Fig 5E–5G and allows a description of the nuclear distribution by just one variable—the position *x*(*t*) of one reference nucleus. (For example, if a given nucleus has coordinates (*x*(*t*), *y*_0_), where *y*_0_ is a constant, its *x*-directed forces come only from the closest neighboring nuclei above and below, which have positions (−*x*(*t*), *y*_0_ − λ) and (−*x*(*t*), *y*_0_ + λ). The two next neighbors located at (*x*(*t*), *y*_0_ − 2λ) and (*x*(*t*), *y*_0_ + 2λ) apply only *y*-directed forces that mutually cancel.) To find the rate of change of the *x*-positions of the nuclei, we simply computed the *x*-component of the force acting on each nucleus by using Eq (1). The resulting equation has the form:

$$\begin{array}{c} \frac{\;dx(t)}{\;dt}=\frac{4f(d(x(t)))}{d(x(t))}x\left(t\right)+g_{\text{S}}\left(b+x\left(t\right)\right)-g_{\text{S}}\left(b-x\left(t\right)\right),\;\text{where}\\ d(x(t))=\sqrt{4x{\left(t\right)}^{2}+\lambda^{2}}. \end{array}$$ (2)

The function *d*(*x*) gives the distance between the reference nucleus and each of its intermediate neighbors. This is an autonomous, first-order ODE for *x*(*t*), which makes it easy to analyze the predicted nuclear positioning. First, note that the SF configuration, *x* = 0, is always a solution of Eq (2). Linear stability analysis of Eq (2) shows that the SF positioning is stable if and only if $2f\left(\lambda \right)/\lambda <-g_{S}^{\prime }\left(b\right).$ By using expressions for the forces between nuclei and between nuclei and cell sides, we found conditions on the model parameters which ensure the SF nuclear order:

$$\begin{array}{c} \frac{b^{1-\alpha_{S}}}{\lambda^{1-\alpha_{N}}}<-\frac{\sigma_{S}\;\alpha_{S}\;M_{S}\;d_{\text{ref}}^{\alpha_{N}-\alpha_{S}}}{2}. \end{array}$$ (3)

Much insight can be obtained from this inequality. First, it showed that to form the SF at all, the nuclear-cell side forces have to be attractive and increasing with distance (as in Model 1), or repulsive and decreasing with distance (as in Model 2), consistent with the fact that the nuclear-cell side forces have to promote centering. From the experimental data we knew that thinner cells and cells with fewer nuclei are more likely to form SFs (Fig 1B, 1F and 1G), that is, the left hand side of Eq (3) has to be an increasing function of parameter *b* and a decreasing function of parameter λ = 2*l*/(*N* + 1), implying that *α*_*N*_ ≤ 1 and *α*_*S*_ ≤ 1. This did not lead to any restrictions for Model 2; however, for Model 1, it showed that the attractive nuclear-cell side forces can at the most grow linearly with distance. Otherwise, very wide cells would form single file rows, in contradiction with the experimental data.

Fig 1B shows the bifurcation value given by Eq (3) in (λ, *b*) space together with the measured data. Any admissible combination of parameters *α*_*N*_ ≤ 1 and *α*_*S*_ ≤ 1 can correctly predict the switch from SF to DF. Thus, the data from the available population of cells were not sufficient to constrain the models further. The most likely reason was that the number of nuclei *N* in the cells correlates with the cell area, and because all cell lengths are similar, with the cell width 2*b*. The models make explicit predictions for future experiments: by forcing the cells into micro-wells and/or extracting nuclei from the cells, one can generate very narrow cells with too many nuclei, or very wide cells with few nuclei. In these cases, the data will contain points in a wider region in (λ, *b*) parameter space, which will allow constraint on the exponents in the models.

The models allowed a prediction of geometric parameters for which we expect SF or DF nuclear arrangements. To make explicit calculations, we returned to the exponents *α*_*j*_ = ±1 used in the screens. For Models 1 and 2, the zero solution *x* = 0 was a steady state of Eq (2), meaning that the SF pattern is always a mechanical equilibrium. This SF pattern is stable, if and only if,

$$\begin{array}{c} \lambda >\sqrt{\frac{2}{M_{S}}}d_{\text{ref}}≕\lambda^{*}\;\left(M1\right),\;b<\sqrt{\frac{M_{S}}{2}}\lambda ≕r^{*}\lambda \;\text{(M2)}. \end{array}$$

At λ = λ* (M1), *b*/λ = r* (M2) the models predicted a pitchfork bifurcation and a DF pattern emerged. Note that Model 1 predicted that the SF pattern is stable if the nuclear spacing along the cell length is greater than the critical value λ*, otherwise the DF pattern emerged. Independence of the DF/SF transition from the cell width in Model 1 was explained by the nuclear-cell side forces scaling with the cell width. Model 2 predicted that the DF/SF transition was determined by the critical ratio r* of the cell width to the nuclear spacing along the cell length. These predictions can be used to interpret future experiments with very narrow cells with too many nuclei, or very wide cells with few nuclei.

We noted that both models predicted a forked bifurcation for the stable *x*-coordinate of the nuclei as a function of the cell width (including Model 1, because in the imaged cells, the average spacing of the nuclei along the *y* axis depends on the cell width). Namely, for the DF pattern, the stable *x*-coordinates for the nuclei in the two files are given by the expressions:

$$\begin{array}{cc} x=\pm \frac{1}{2}\sqrt{{\left(\lambda^{*}\right)}^{2}-\lambda^{2}},\;\lambda \;<\;\lambda^{*}\;\left(M1\right); & x=\pm \sqrt{\frac{b^{2}-{\left(r^{*}\lambda \right)}^{2}}{2M_{S}+1}},b/\lambda \;> \end{array}\;r^{*}\;\left(M2\right).$$ (4)

To compare these predictions with the data, we found the force magnitude *M*_*S*_ by minimizing the error of the predicted average nuclear *x*-positions per cell, and plotting the result as a function of cell width (Fig 6D). Both models predicted the bifurcation from SF to DF pattern observed in the data very well.

#### Nuclear shapes

The nuclei in the imaged VL3 and VL4 muscle cells have ellipsoidal shapes (Fig 1B and 1D). Using the ellipses’ orientation angle *φ* ∈ [0, *π*) (direction of the major axis) and the lengths of the major *l*_*M*_ and minor *l*_*m*_ axes, we defined the aspect ratio (AR) of a nucleus, *N*_AR_, as *l*_*M*_/*l*_*m*_ for |*φ* − *π*/2| ≤ *π*/4, and as its reciprocal value otherwise. In other words, the aspect ratio is greater/less than 1 if the nucleus is oriented along/normal to the cell long axis, respectively (Fig 6F).

We assumed that the nuclei are deformable viscoelastic bodies [32] and that their ellipsoidal shapes resulted from the anisotropy of the net stress applied to each nucleus by the positioning forces. We denoted by *f*_*x*_ and *f*_*y*_ the forces acting on the nucleus from each side in *x* and *y*-direction respectively (positive for pushing, negative for pulling forces). We further assumed that the forces from the left and right lead to a change in nuclear width by a factor 1 − *f*_*x*_/*κ*, where *κ* is a constant describing the nucleus’ resistance to deformations and is proportional to its Young modulus. We next assumed the nuclear area is conserved, so the same forces from the left and right cause a change in nuclear height by a factor 1/(1 − *f*_*x*_/*κ*). Applying the same argument to deformations caused by forces from above and below, we approximated the nuclear aspect ratio as:

$$\begin{array}{c} N_{\text{AR}}=\frac{{\left(\kappa -f_{y}\right)}^{2}}{{\left(\kappa -f_{x}\right)}^{2}}. \end{array}$$ (5)

We used the equations determining the forces between the nuclei and between the nuclei and cell sides and the nuclear equilibrium positions to calculate the left-right and up-down forces *f*_*x*_ and *f*_*y*_. The question arose whether we should include only interactions between the nearest neighbors to a given nucleus, or the second neighbors directly above and below the nucleus as well. We considered both options, calling them *short range* and *long range*. Denoting by $\bar{x}$ the equilibrium position for DF as given by (4), the forces *f*_*x*,*y*_ are given by the formula:

$$\begin{array}{cc} & SF:f_{x}=2g_{S}\left(b\right),\;f_{y}=2f\left(\lambda \right) \end{array}$$

$$\begin{array}{cc} & DF:f_{x}=g_{S}\left(b-\bar{x}\right)+g_{S}\left(b+\bar{x}\right)+4\frac{f(d\left(\bar{x}\right))}{d(\bar{x})}\bar{x},\;f_{y}=\{\begin{array}{c} 2\frac{f(d\left(\bar{x}\right))}{d(\bar{x})}\lambda ,\;\text{for}\;\text{short}\;\text{range}\\ 2\frac{f(d\left(\bar{x}\right))}{d(\bar{x})}\lambda +2f\left(2\lambda \right),\;\text{else.} \end{array} \end{array}$$

For each variant of Models M1 and M2 (short and long range), we applied Eq (5) to all 200 imaged cells (geometric parameters of each cell were substituted into the formula to evaluate *f*_*x*_ and *f*_*y*_ forces). We found parameter *κ* by minimizing errors to find the best fit to the measured nuclear aspect ratio. Fig 6E depicts the outcome as a function of cell width. In Model 1 attractive forces between the nuclei and cell sides elongated the nuclei toward the sides, and repulsive forces between the nuclei flattened the nuclei from the poles. On the other hand, in Model 2 with long range nuclear forces, predicted the nuclear aspect ratio remarkably well. The repulsion from the sides overcame the repulsion from other nuclei above and below, and ultimately elongated the nuclei along the cell long axis.

These results suggest Model 2 as the most appropriate one; it was able to explain well all available data. In addition, this model was the most robust, as the patterns which it predicts are least sensitive to parameter variations.

### Comparison to agent-based, stochastic simulations

The screen of the interacting particle models resulted in a single model that fits the data best. According to this model, the nuclei repell each other and the cell boundaries with the long-range forces that decrease with distance. This strongly suggested that pushing of MTs, either by polymerization, or by kinesin-generated force on the MT plus ends, establishes nuclear positioning: the number of growing MT ends decreases with the distance from the nucleus, and we expect a repulsive force decreasing with distance in this simple scenario. To test this model in a concrete molecular context and confirm the assumptions A3-5 that underline the usage of deterministic, isotropic, and distance-dependent forces in the interacting particle model (Sec Force-balance and force-screening), we turned to a detailed agent-based simulation of microtubule-generated mechanics of the multi-nuclear cell. Such a model allowed examination of whether stochastic effects are negligible, and whether MT bending and resulting elastic forces result in unforeseen effects.

We choose to use the microscopic, stochastic simulation tool Cytosim [24, 33], which has been successfully applied to a wide range of cell biological problems [34–36]. Using Cytosim, we simulated hundreds of MTs per nucleus, which are distributed uniformly around the nuclear circumference, cantilevered in the nuclear envelope and grow in a radially symmetric way. Individual MTs are treated as elastic rods, with the length of each MT characterized by the stochastic dynamic instability process [29], whereby each MT undergoes repeated stochastic cycles of growth, catastrophe, shortening and rescue. Contact of a growing MT end with a neighboring nucleus or cell boundary results in MT bending, which generates an elastic pushing force. While pushing forces do not decrease the growth rate of MTs, catastrophy rates can increase to a maximum of 0.02 per second (see Tab 2), resulting in relatively brief, but typically more than 50s long, force generation events. Similar force events would be observed in stochastic simulations of forces exerted by a kinesin plus-end motor. Thus we did not include explicit molecular descriptions of kinesins in these simulations. The sum of pushing forces from all MTs constitutes the net force.

**Table 2:** Parameters in the agent-based, stochastic simulation using Cytosim.

Parameter	Values used	Comment
**General**		
time step	0.1*s*	
viscosity	0.05 *pN* *s*/*μm*^2^	order of magnitude from [35]
cell width	40*μm*, 100*μm*	corresponding to a VL4 and VL3 cell
cell height	210*μm*	roughly half the height of real muscle cells
number of nuclei	7	roughly half the number of real muscle cells
initial positions	random	
**Nucleus**		
radius	7*μm*	measurements
MTs per nucleus	200	estimated
**Mictotubuli**		
rigidity	25 *pN* *μm*^2^	[38]
growing speed	0.13 *μm*/*s*	[38]
shrinking speed	0.27 *μm*/*s*	[38]
catastrophe rate	0.005/*s*, 0.02/*s*	in the absence of force, of the stalled tip, order of magnitude from [38]
rescue rate	0.064/*s*	[38]
total polymer	25*μm* per MT	this limits the availability of free tubulin, estimated
growing force	1.7 *pN*	[24]

#### Calculating distance-dependent forces from agent-based simulations

**Fig 7:**
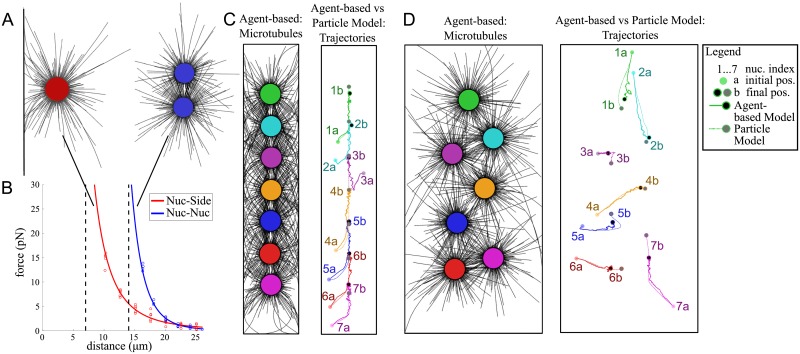
Microscopic simulation using Cytosim. A: To study the force acting on a nucleus created by pushing microtubules, we placed a nucleus near a boundary (left) and near a second nucleus (right) and used the speed to calculate the force (details in Methods). B: Force calculation results from 5 realizations at 7 distances of A (dots) and power law fits of their average. Using the notation of Eq (1) this yielded *α*_*N*_ = −7.6, *α*_*S*_ = *α*_*P*_ = −3.3, *M*_*s*_ = *M*_*p*_ = 12.4, vertical dashed lines mark the radius and diameter of a nucleus. C and D: Comparison between the microscopic simulation using Cytosim and the trajectories of positioning nuclei obtained from solving Eq (1) using the parameters obtained from B. Left: Final positions of nuclei (colored) and microtubuli (black) at *t* = 17*h*, right: Initial positions (transparent, marked with *a*, trajectories (solid and dotted lines) and final positions (marked with *b*, black/grey) of the agent-based and interacting particle simulations. Shown are simulations for a VL4 type cell (C) and a VL3 type cell (D). Movies comparing the simulations are shown in S1 and S2 Video. Parameters of the stochastic agent-based simulation can be found in Table 2, those of the interacting particle simulation are given by the fit in B.

We used the microscopic model, first, to simulate the interactions of a nucleus with a cell boundary in isolation. We placed a nucleus at a set distance from the single cell boundary (Fig 7A left), allowed the MT dynamics to establish for a time period, and then released the nucleus, which started to move away from the boundary. Repeating this experiment for different distances, we used the resulting nuclear speeds to calculated the net force as a function of nuclear distance (Fig 7B). To confirm that this procedure yields correct values, we compared the obtained forces to analytical approximations for the case of a single pushing filament. We found very good agreement between both methods (see S1 Text for details). Second, we simulated the interactions of two nuclei in isolation. We placed nuclei at different distances from to each, let the MTs equilibrate and released the nuclei (Fig 7A right). From the speed of the nuclear divergence, we computed an effective distant-dependent force (Fig 7B). We found that the forces in both cases rapidly decrease with distance (for details see Methods). It should be noted, however, that also more complicated, non-monotonic force-distance relationships have been observed [37], which can affect the equilibrium positions of the nuclei. The precise behavior will depend on the MT dynamics and will have to be revisited when more details about muscle cell MTs become available.

#### Agent-based and interacting particle simulations match closely

In a multinucleated cell, the assumption of pair-wise additive forces could be violated through a variety of factors. For example, if three nuclei are located along a straight line, the nucleus in the middle will, most likely, block direct interactions between the two outer nuclei (shielding effect). To test the importance of possible non-pair-wise interactions and the validity of the particle interaction model, we simulated the positioning mechanism in a typical VL3 and VL4 cell. We used random initial positions and actual cell widths, but only half the typical cell length and number of nuclei. This maintained the ratio of cell length and number of nuclei observed in the experimental data, while saving computational time. Fig 7C and 7D shows that the simulated nuclear patterns are as observed in the experiment and predicted by the simple particle interaction model using pairwise forces, with single and zigzagged double-file spanning the cell length in narrow and wide cells, respectively. Moreover, the trajectories of individual nuclei from their initial positions to the final, equilibrium positions were strikingly similar in the microscopic agent-based model and the particle interaction model (Fig 7C and 7D). For the latter, we used the distance-dependent forces calculated from the stochastic nucleus-boundary and nucleus-nucleus simulations to inform the values of the force exponents and force magnitudes in Eq (1). The striking similarity between the stochastic and deterministic trajectory validated the simple particle interaction model. Further these results suggest that, due to the rapid decrease of the net force with distance, non-pairwise interactions and possible shielding effects are negligible.

## Discussion

In this study, we used a “big” imaging data set generated from 2500 myonuclei from *Drosophila* larvae to computationally examine potential mechanisms of positioning of multiple nuclei in the cells. Rather than employing a traditional reductionist modeling approach, we used a different modeling philosophy, sometimes called “ensemble modeling” or “reverse engineering”. The idea is to start with multiple possible models and to use the (predicted) data to eliminate as many models as possible, dependent on their ability to recapitulate biological systems. This approach was successfully applied to cell signaling dynamics, metabolic networks, cell cycle, and spindle geometry [28, 39–41]. In some instances, a small number of models can be analyzed one by one, as in a recent study on chemotaxis model inference [42]. In other instances, the number of model variants is so great that an unsupervised or semi-supervised computer screen of the models is necessary [28].

We searched computationally for the types of forces that could occur between pairs of nuclei and between nuclei and the cell boundary and could lead to positioning of the myonuclei. A similar problem, mitotic spindle positioning, has a long history [21] and reductionist modeling proved helpful in that case. However, an approach philosophically similar to ours was recently applied successfully to search for forces positioning the sperm MT aster in sea urchin eggs [43].

We started with a large number of potential forces and formulated a few hundred potential models, each characterized by a few mechanical parameters. We then used 1.5 million simulations to solve the differential equations describing movements of the nuclei predicted by each of these models at various parameters. We filtered out the vast majority of the models that were not able to predict the uniform spread of the nuclei along the cell long axis or the tendency of the nuclei to self-organize into the single file in narrow cells and double file in wide cells. The remaining 12 model classes were further tested by their ability to predict correct average nuclear position along the short axis of the cell and on robustness, the relative insensitivity of the models’ behavior to parameter values. These tests left us with two possible models, the parameters of which were fully determined by requiring the models to quantitatively fit the data in 200 imaged cells.

The remaining two models made three non-trivial predictions: 1) the double-file pattern in wide cells is a zig-zag; 2) the average nuclear position along the cell short axis has the forked bifurcated dependence on the cell width, and 3) nuclear density is higher near the cell poles. Note that both models closely fit the experimental data very well, even though these data was not used to find the models’ parameters. Remarkably, these two models make opposite predictions about the nuclear shapes. One of the models predicts that the nuclei have ellipsoidal shapes with the long axes oriented perpendicular to the cell long axis, which is contrary to the experimental data. Incidentally, this model is also less robust than the other, ultimate, model, which not only predicts correctly that the ellipsoidal nuclei have long axes oriented along the cell long axis, as observed, but also fits very well the measured dependence of the nuclear aspect ratio as function of the cell width.

Ultimately, only one model recapitulates all characteristics of nuclear positioning in VL muscle cells. It suggests that, nuclei repel each other and the cell boundary with forces decreasing with the distance. Our data suggest a simple molecular mechanism, which generates MT pushing forces, either by MT polymerization, or by MT interactions via kinesin motors on the nuclear envelopes and cell cortex.

We support the computational screen of the simple models, in which the nuclei interact as particles by isotropic and deterministic forces, with simulations of a detailed agent-based mechanical model, in which we simulate hundreds of MTs undergoing dynamic instability, bending and pushing on the nuclei and boundary with elastic forces. These simulations support the types of forces hypothesized in the simple models, confirming that subtle stochastic, elastic and geometric effects do not invalidate the simple models’ assumptions. More importantly, the agent-based simulations generate the single- and double-file nuclear patterns in narrow and wide cells, respectively, as observed and as predicted by the simple models. Note that each simulation of the microscopic model took hours up to many days on an Linux machine with a Intel Core i7-7700 processor. As such, parameter exploration of the detailed models, or testing whether they reproduce subtle observed data features, is prohibitive. In the future, we plan to use more sophisticated mathematical methods [44] of solving the inverse problems—inferring the models from the data.

While the involvement of MTs and molecular motors in the nuclear positioning is firmly established, we do not provide direct proof that a mechanical force balance is the main mechanism of nuclear positioning. Another possibility is that there is a preexistent, perhaps morphogen-governed, pattern in the cell, and that MTs simply tether the nuclei to special locations in this pattern. Relevant to this thought is the fact that small nuclear clusters aggregate at neuromuscular junction in mammalian cells. Our model makes detail predictions about nuclear positions in resting/fixed muscle cells. However, functioning muscle cells contract, and it is likely that the actomyosin contraction forces are orders of magnitude greater than the MT-based forces. Thus, it is hard to imagine that MT asters are sufficient to resist nuclear displacement during muscle contractions, and additional nuclear tethers might be involved in maintainaing an established pattern [15]. Future *in vivo* experiments, including genetic and biophysical manipulations and live cell imaging, will be required to investigate nuclear positioning in contracting muscle cells. However, we note that our model generates specific, testable predictions about the nuclear pattern in cases where the cells acquire unusual shapes and sizes or contain variable numbers of myonuclei.

Another intersting aspect of muscle biology that could benefit from our modeling approch is the initial positioning of nuclei in developing embryonic muscle fibers. In the early embryonic muscle cells in *Drosophila*, after myoblast fusion, the nuclei initially cluster together, then split into two clusters that segregate to the cell poles, and finally spread along the cell length [16]. It remains to be tested if a force balance model can explain these dynamics. Even more challenging is the problem of coupling of the cell growth, shape change, and protein synthesis with the dynamics of nuclear numbers, positions, sizes and transcriptional activity. Last, but not least, the majority of the muscle cell types are cylindrical, with nuclei positioned at the cell periphery on all the cell’s surfaces. These essentially 3D nuclear patterns require special studies.

Active, non-random nuclear positioning has been attracting increasing attention lately [45]. In a number of recent studies, force generated by MTs and motors were shown to be crucial for nuclear positioning and movement [24, 46, 47]. We suggest that the approach that we describe here—sequential computational screen of particle interaction models followed by detailed agent based simulation of the force balance model—is the optimal way to incorporate modeling as part of the experiments directed at understanding not only multi-nuclear positioning in mammalian muscle cells, but also in syncytium, giant cells, granulomas and osteosarcomas, as well as mechanisms of other organelles’ positioning.

## Methods

### Fly stocks, dissections, labeling, imaging

The following *Drosophila* stocks were maintained at standard conditions on cornmeal medium: *w*^1118^ (Bloomington 3605), *Dmef2-GAL4* [48], *UAS-2xEGFP* (Blomington 6874), *UAS-GFP RNAi* (J. Zallen, SKI). Crosses (UASxGAL4) were performed at 25°C; embryos hatched within a 2h period were selected and raised to third instar larval stage. Wandering third instar larvae were dissected and fixed in 10% formalin and labeled as previously described [16]. Muscle cells were labeled using Alexa Flour-conjugated phalloidin (Life Technologies); nuclear DNA was visualized with Hoechst 33342 (Invitogen). Anti-Lamin (DHSB, ADL 67.10) and anti-*α*-Tubulin (Sigma, T9026) primary antibodies, and Alexa Flour-conjugated secondary antibodies (Life Technologies) were used to label the nuclei and microtubules, respectively. Whole larvae were mounted in ProLong Gold antifade reagent (Invitrogen). VL3 and VL4 muscles in abdominal hemisegments 2-6 were imaged on a LSM 700 confocal microscope (Zeiss).

### Image processing and quantification

Quantification of confocal *z*-projections was performed using standard ImageJ and Matlab measurement tools. VL3 and VL4 cells were traced by hand, based on phalloidin labeling. Automated thresholding of fluorescence intensities of anti-Lamin and/or Hoechst labeling was used to generate binary images of VL nuclei. Nuclear centroids were used to calculate nearest neighbor distances. Cell widths and lengths heights were defined as average widths and lengths of the measured boundary. Nuclear (*x*, *y*) positions were transformed onto positions in a rectangle using a mapping that preserves relative distances from the boundary. ImageJ’s ellipse fitting tool was used to define nuclear shapes. Nuclear pattern of both experimental and simulated origins were categorized into *single file* (SF), *double file* (DF) or *neither* using a histogram of the relative nuclear *x*-positions with 7 equally spaced bins. If the histogram had only one peak containing at least 60% of all nuclei, the cell was classified as SF. If the histogram had two peaks which together contained at least 60% of all nuclei, and the number of nuclei they contained differed by less than 50%, the cell was classified as DF, in all other cases as neither.

### Filter 1

Parameters are shown in Table 1. Simulation details are given below, *r* = 7*μm* is the radius of a nucleus. The criteria K1-4 for valid patterns are as follows (referring to Fig 3 △ = K1, ◻ = K2, ▼ = K3, ○ = K4, ◼ = K5). K1: All nuclei centroids have to be at least *r* away from all cell sides and poles. K2: All nuclei centroids have to be at least 2*r* apart. K3: The nuclear pattern is classified as DF or SF for the wide and thin cell respectively (see DF/SF classification details). K4: max *y* − min *y* > 2/3×cell height. K5: The mean nearest neighbor distance is larger than 30*μm* and 45*μm* for the wide and thin cell respectively. The last criterion avoids counting random patterns as false-positive (compare Fig 1E). Candidate models have to lead to valid patterns in both cell geometries, but not for the same parameters (this avoids missing good models).

### Filter 2

Parameters are shown in Table 1. Simulation details are given below. The criteria for valid patterns are similar to Filter 1, however since cells of variable width and number of nuclei were used, the SF/DF criteria is dropped and criteria K5 is replaced by K5^⋆^: We required that the mean NND of all nuclei (in all 14 cell geometries) is 40 ± 5*μm*. For each model, we take only parameter combinations leading to valid patterns (K1-K4,K5^⋆^) and minimize the mean error of the predicted average *x*-position as a function of cell width (compared to the measured behavior). The curves shown in Fig 4A correspond to the parameters that minimize that error. Robustness with respect to the force range (Fig 4B) is obtained as follows: For each model and each combination of force ranges *c*_*N*_, *c*_*S*_, for each value of *M*_*S*_ the fraction of the 14 cells fulfilling criteria K1-K4,K5^⋆^ was determined, then added for all four criteria and finally maximized over the values of *M*_*S*_. This yielded a score between 0 and 4 for each model and pair (*c*_*N*_, *c*_*S*_), the color in the Fig 4B represents this score.

### Correlation analysis

Only SF and DF cells (see above) were used and nuclei within 10% cell height of the poles were disregarded. The remaining $\hat{N}$ *y*-positions in each cell were normalized via $y_{\text{norm}}=\frac{y}{\max y-\min y}\left(\hat{N}-1\right)$ (if the *y*-positions were equally spaced, this would yield a *y*-spacing of exactly 1) and all positioned were shifted, such that the middle-most nucleus has *y*-position zero. Now the normalized *y*-positions of all cells were collected (using all SF *y*-positions for the SF auto-correlation analysis, and separating *y*-positions of nuclei right, and left of the middle of the cell for the DF correlation analysis). For the final histograms a bin spacing of 0.25 was used.

### Simulation: Interacting particle model

To determine equilibrium positions, Eq (1) was solved on a rectangular domain using Matlabs ode solver *ode15*, a variable-step, variable-order solver. To model finite size effects of nuclei, a size exclusion term was added in Eq (1). For two nuclei whose centroids are a distance *d* apart, it takes the form

$$\begin{array}{c} f_{\text{SE}}=Q_{\text{SE}}(\frac{1}{d^{2}}-\frac{1}{{\left(2r\right)}^{2}})H\left(2r-d\right), \end{array}$$

where *r* = 7*μm* is the nuclear radius, *H* is the Heaviside function and *Q*_SE_ = 2000 describes the strength of the size exclusion. For size exclusion effects between nuclei and the cell boundary, 2*r* was replaced by *r*. Simulations were run until all of the right-hand-sides in Eq (1) had a 2-norm of less than 10^−4^. Codes are available upon request.

### Simulation: Stochastic agent-based model

The simulation software Cytosim (Ver. 3, 2007) [26, 33] was used. The configuration files are available upon request. To calculate a (distance dependent) force from a measured nuclear speed *v* we used the effective viscosity of the aster *η*_eff_ consisting of the sum of the nuclear and MT drag as implemented in Cytosim. The force is then given by *f* = *v* *η*_eff_.

## Supporting information

S1 Text This file contains three sections: *1. Effect of internuclear friction.* This section describes the modeling and simulation of nucleus-nucleus friction. *2. Attraction-repulsion internuclear forces.* This section explores the implications of internuclear forces that change character (attractive/repulsive) at some distance in a simplified 1D situation. *3. Comparison of numerical and analytical results for MT-mediated forces.* This section compares the shape of a single clamped, confined microtubule, as well as the forces it creates as computed by Cytosim and using an analytical approximation.
(PDF)

S1 Video Agent-based and interacting particle model—Thin cell. The video compares the agent-based, stochastic simulations in Cytosim (left) with an interacting particle simulation (right) in the thin, VL4 type cell. Microtubuli are shown as white lines, nuclei as red solid circles, the distance dependent force in the interacting particle model is symbolized by shades of red and yellow.
(AVI)

S2 Video Agent-based and interacting particle model—Wide cell. The video compares the agent-based, stochastic simulations in Cytosim (left) with an interacting particle simulation (right) in the wide, VL3 type cell. Microtubuli are shown as white lines, nuclei as red solid circles, the distance dependent force in the interacting particle model is symbolized by shades of red and yellow.
(AVI)
