# Supplementary material for: Mechanical positioning of multiple nuclei in muscle cells
Source: PLoS Comput Biol. 2018 Jun 11;14(6):e1006208. doi: 10.1371/journal.pcbi.1006208 (PMC6013246; doi:10.1371/journal.pcbi.1006208)
Supplement: S1 Text — This file contains three sections: 1. Effect of internuclear friction. This section describes the modeling and simulation of nucleus-nucleus friction. 2. Attraction-repulsion internuclear forces. This section explores the implications of internuclear forces that change character (attractive/repulsive) at some distance in a simplified 1D situation. 3. Comparison of numerical and analytical results for MT-mediated forces. This section compares the shape of a single clamped, confined microtubule, as well as the forces it creates as computed by Cytosim and using an analytical approximation. (PDF) [file pcbi.1006208.s001.pdf]

# Mechanical positioning of multiple nuclei in muscle cells - Supplementary Information

Angelika Manhart[1], Stefanie Windner[2], Mary Baylies[2], Alex Mogilner[1,*]

1. Courant Institute of Mathematical Sciences,
New York University, 251 Mercer Street,
New York, NY 10012, USA

2. Program in Developmental Biology,
Sloan Kettering Institute, Memorial Sloan Kettering Cancer
Center, New York, New York 10065

* Corresponding author: mogilner@cims.nyu.edu

## 1 Effect of the Internuclear Friction

In the coarse-grained, interacting particle model we assumed that the velocity of each nucleus is proportional to the total force applied to this nucleus divided by an effective constant viscous drag. However, an entanglement of dense microtubule (MT) networks growing from neighboring nuclei, and/or MT associated protein-mediated interactions of these networks could lead to an effective nucleus-nucleus friction, as has been suggested e.g. in [1]. To test implications of this possible internuclear friction, we formulated and simulated the following simple model.

**Model with internuclear friction.** If we denote by $F_i \in \mathbb{R}^2$ the total force acting on the $i^{th}$ nucleus (i.e the right-hand-side of Eq (1) in the main manuscript), then the equations of motion of this nucleus, which take into account both nucleus-nucleus and cytoplasmic frictions, read:

$$\frac{\mathrm{d}X_i}{\mathrm{d}t} = V_i \tag{1}$$

$$V_i = F_i - \delta \sum_{\substack{j=1 \\ j\neq i}}^{N} \left[\chi(\|X_i - X_j\|)\,(V_i - V_j)\right], \qquad i = 1, .., N.$$

In this model, the internuclear friction is a function of the difference of the velocities between neighboring pairs of the nuclei, such that each nucleus effectively drags other nuclei in its vicinity in the same direction as its own motion vector. Thus, the second

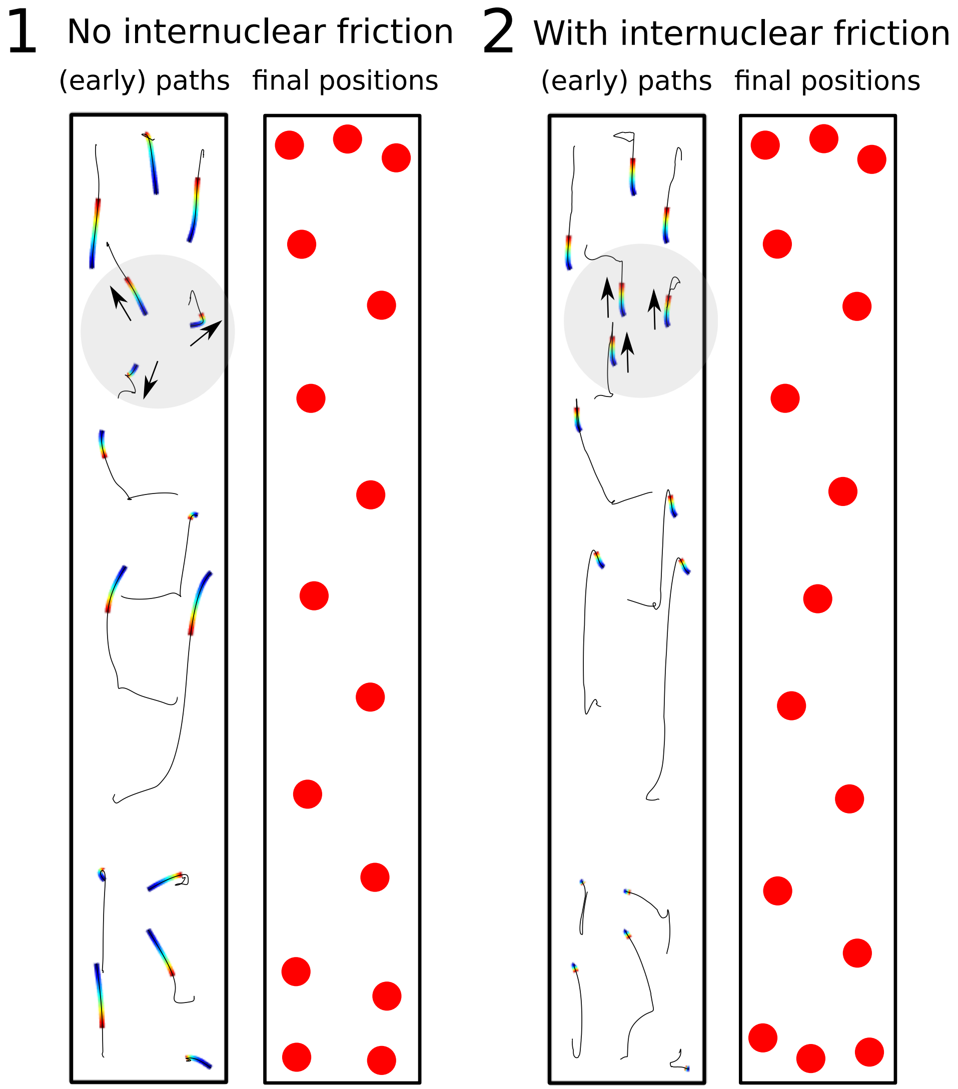


Figure A: *Effect of internuclear friction.* 1: No internuclear friction, i.e. setting $\delta = 0$ in (1), which corresponds to Eq (1) in the main manuscript. 2: Strong internuclear friction, using $\delta = 10$ in (1). For both simulations the same random initial positions were used. Left: The nuclei paths during the simulation, black-thin, and the initial movement, colored-thick, from early (blue) to later (red). The grey circle highlights three nuclei (see text), arrows emphasize their initial movement. Right: Final equilibrium positions, red dots.

equation is, in fact, a linear algebraic system that determines the velocities of all nuclei for each configuration of the system. Parameter $\delta$ is the ratio between the internuclear and cytoplasmic friction, while the function $\chi(d)$ determines the friction magnitude between a pair of nuclei at distance $d$ from each other. For simplicity, we choose $\chi(d) = 1$ for $d < d_{\text{neigh}}$ and zero otherwise, i.e. the nuclei affect each others speed only if they are closer than the threshold distance $d_{\text{neigh}}$. Numerically, we determine the velocities at each time step by solving the second equation in (1), i.e. the liner system for the velocities $V_i$ $i = 1, .., N$ by calculating the values of the forces $F_i$ and of the function $\chi$ from the

nuclear positions determined in the previous time step.

**Internuclear friction affects the transient dynamics significantly, but the final positions only a little.** As preliminary test we simulated the effect of internuclear friction on the positioning for the final model M2 (all forces repulsive, decreasing with distance). We used M2 model parameters $M_S = M_P = 1$ and set $d_{\text{neigh}} = 100\mu m$ and $\delta = 10$, i.e. a relatively strong effect of internuclear friction. Fig A depicts the outcome. As can be seen in Fig A1-2 right, the final positioning pattern, though not identical, is very similar to that in the model without the internuclear friction. However, initially the paths of the nuclei in the model with the internuclear friction, shown in Fig A1-2 left, are much more correlated to those in the model without the internuclear friction. For example, the three nuclei marked by the grey circle, initially move away from each other without internuclear friction, but move in the same direction, if internuclear friction is included.

While systematic understanding of the effects of an effective internuclear friction requires further exploration, this suggests that, since this study is concerned with equilibrium positioning only, it is justified to disregard internuclear friction. In the future, time-series data on the nuclear movement can be used to assess the influence of internuclear friction on mynuclear movements.

# 2 Attraction-repulsion internuclear forces

In the force screen with the coarse interacting particle model, we restricted ourselves to forces that are either repulsive or attractive at all distances. Here we investigate the effect of forces that at some distance switch from attraction to repulsion. For simplicity we asses only if and how the nuclei spread along the long axis of the cell, i.e. we consider a 1D model along the $y$-axis. This means that we omit any side forces, and we also assume that there are no pole forces. Here, we consider internuclear forces of the form

$$f(d) = \left(\frac{d}{d_{\text{ref}}}\right)^r - A\left(\frac{d}{d_{\text{ref}}}\right)^a. \tag{2}$$

As in the main text, $d_{\text{ref}}$ is a fixed reference distance. The exponents $r$ and $a$ can be positive or negative and describe the behavior of the repulsive ($r$) and attractive ($a$) force components with respect to distance. Finally, $A > 0$ measures the magnitude of the attractive forces compared to the repulsive ones. As in the main model, we add a size-exclusion force (now shown) between pairs of nuclei and nuclei and the poles. A similar model has been investigated in [2] in the context of social aggregation.

We consider the following four most likely cases, depicted in the first row in Fig B:

1 Short ranged repulsion, long ranged attraction with attraction increasing with distance, e.g. $r = -1$, $a = 1$;

2 Short ranged repulsion, long ranged attraction with attraction decreasing with distance, e.g. $r = -2$, $a = -1$;

3 Short ranged attraction, long ranged repulsion with repulsion increasing with distance, e.g. $r = 1$, $a = -1$;

4 Short ranged attraction, long ranged repulsion with repulsion decreasing with distance, e.g. $r = -1$, $a = -2$.

**Attraction-repulsion forces lead to varying nuclear patterns.** We screened the model behavior for all cases with respect to the parameter $A$. We simulated the 1D

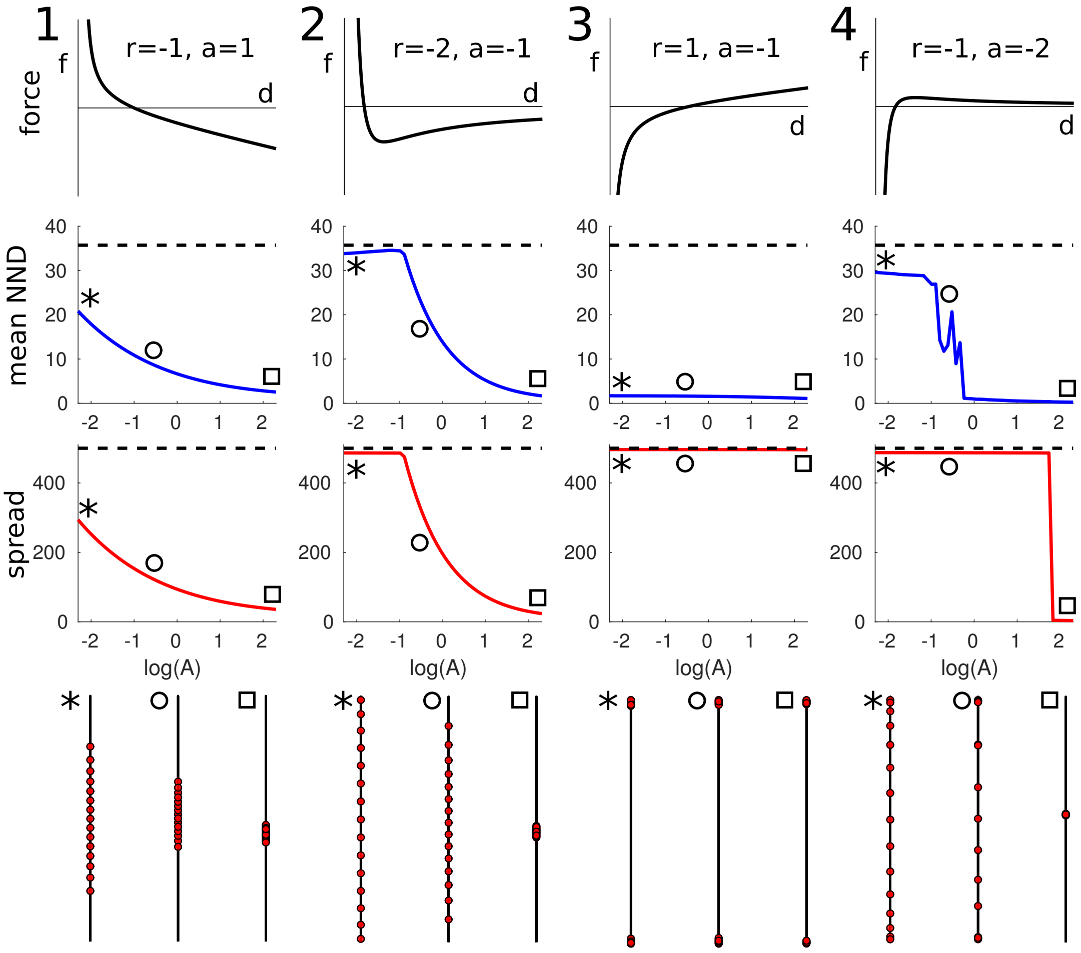


Figure B: *Attraction-repulsion internuclear forces.* Columns 1-4: The four cases described in the text. First row: Internuclear forces $f$ as functions of distance $d$, the values of the exponents refer to (2). Second and third rows: The mean nearest neighbor distance (NND) (second row) and maximal spread along the axis (third row) of the equilibrium patterns as functions of $\log(A)$. The dashed lines mark the case of perfect equidistant spacing along the whole cell. The symbols mark the values of the parameter $A$ for which example patterns are shown in the fourth row. Fourth row: Example equilibrium patterns for the four force types and for the values marked in the second and third row.

system for random initial conditions and $N = 15$ nuclei in a cell of height $2l = 500\mu m$ using $A \in [0.1, 10]$. We evaluated the resulting equilibrium patterns by calculating two pattern descriptors: the mean nearest neighbor distance (NND) (Fig B second row) and the maximal spread along the $y$-axis (Fig B third row). Perfect equidistant spacing along the whole cell would yield a spread of 500 and a mean NND of $2l/(N-1) = 35.7$, while all nuclei in a single cluster would yield a spread and mean NND of zero.

For case 1, we found that the resulting pattern is always a roughly equally spaced group that gets more and more condensed as $A$ increases, but never fully collapses. Collapse is prevented by the large short-ranged repulsive forces, while the increasing attractive forces at long distances cause the compactification of the whole group. In case 2, equal spreading along the full axis is achieved for small values of $A$, however at attraction higher than critical, the group starts to become more condensed. This behavior has already been observed in [2] where it was noted that in the case of strong attraction, more particles will not increase the group size, but rather lead to more crowding. In case 3, the nuclei form two clusters at the poles, irrespective of the parameters. This is a simple consequence of the large, small-ranged attraction (promoting clustering) and the strong repulsion at large distances (driving the clusters apart). Finally in the case of 4, nuclei spread equally along the full axis for small values of $A$; if $A$ increases further, clusters begin to form. Their number decreases until all nuclei collapse into one cluster. In this case, the internuclear forces create an unstable equilibrium at the distance $d = Ad_{\text{ref}}$. Nuclei that are initially closer than this distance, will be attracted to each other and form a cluster. The larger $A$, the larger this sphere of attraction will be, hence fewer clusters are created. It is important to note, that even for small values of $A$, pairs of nuclei that are very close initially, will create a small cluster, i.e. the dynamics cannot move them apart.

**Main outcomes of the force screen are not affected by the attraction-repulsion forces.** In summary, we found that for short ranged repulsion (cases 1 and 2), small amounts of attraction only create a small perturbations of the equidistant nuclear pattern. On the other hand, the main screen excluded all attractive internuclear forces. The above simulations (cases 3 and 4) show that adding repulsion at larger distances does not prohibit the clustering, hence the main conclusions of the force screen are not affected.

# 3 Comparison of numerical and analytical results for microtubule-mediated forces

In the main text, we reported the results from using the stochastic, agent based simulation tool Cytosim [3] to calculate average forces created by an aster of stochastically growing and shrinking microtubules (MTs). The resulting force on the nucleus is a consequence of the bending stiffness of the attached MTs. For the case of microtubules of a fixed length, these forces, as well as the MT shapes can be approximated analytically [4], a procedure which has been used e.g. to determine equlibrium positions of the spindle in a dividing cell [5]. Here, we use the analytical formulas to compare the resulting calculated MT

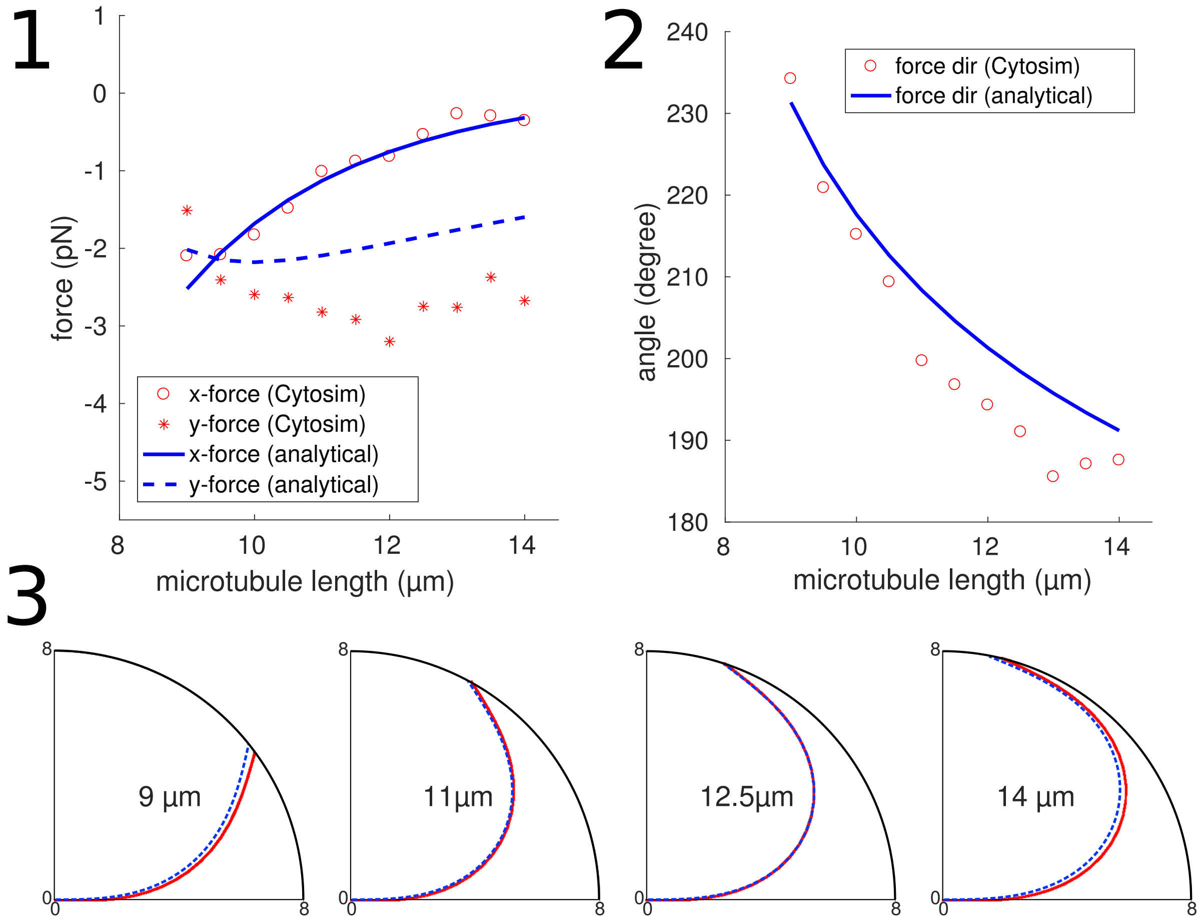


Figure C: *Comparison of the numerical and analytical approaches.* 1: Forces on the nuclear attachment point for various MT lengths calculated by using the analytical expressions given in (4) (blue lines) and by using Cytosim (red symbols). 2: As in 1, but showing the force directions (measured from the top, clock-wise). 3: As in 1 and 2, but showing the MT shapes for four different MT lengths computed by solving (3) (blue-dashed) and by using Cytosim (red-solid).

shapes and forces to those computed using Cytosim.

We consider a single MT with bending stiffness EI=25 $pN \cdot \mu m^2$ of length $L$, positioned in a disk of radius $R = 8\mu m$. One MT end is fixed at the center, and its direction is clamped to 90° (all directions are measured from the top, clock-wise). Let $s \in [0, L]$ be the arc-length along the MT, and $(x(s), y(s))$ the position of the corresponding material point ($s = 0$ is at the nuclear envelope). We use an arc-length parametrization and write: $\dot{x}(s) = \sin(\theta(s))$, $\dot{y}(s) = \cos(\theta(s))$, where the direction $\theta(s)$ can be found from the standard equations of elasticity theory [6]:

$$\begin{aligned} &\ddot{\theta}(s) = \frac{P}{EI}\sin(\beta - \theta(s)), \quad \theta(0) = \pi/2, \quad \dot{\theta}(0) = 0 \\ &x(0) = 0, \quad y(0) = 0, \\ &x(L) = R\sin(\beta), \quad y(L) = R\cos(\beta). \end{aligned} \tag{3}$$

In this system, the force magnitude $P$ and the direction of the MT tip $\beta$ are unknown parameters determined from the solution of the problem. We use the following analytical approximation for these two parameters given in [4]. Let $\varepsilon = (L - R)/L$, then:

$$P = EI\frac{\pi^2}{L^2}\frac{1 + 0.09\varepsilon^3}{1 - 0.5\varepsilon}, \tag{4}$$

$$\beta = \pi/2 \pm \frac{2\sqrt{\varepsilon}}{1 - \frac{5}{48}\varepsilon - \frac{1}{53}\varepsilon^2}.$$

Note, that there are two solutions corresponding to MT bending in the clock-wise and counter-clock-wise directions; in the following, we always use the counter-clock-wise one. The resulting force, $F = (f_x, f_y)$, on the point connected to the nuclear envelope is now given by:

$$f_x = -P\sin(\beta), \quad f_y = -P\cos(\beta).$$

Fig C1 and 2 show the force components and force direction as functions of the microtubule length $L$, and Fig C3 (blue lines) shows the resulting MT shapes.

Finally, we compare the shapes and forces obtained analytically with those obtained in an equivalent assay in Cytosim. In this case, we calculate the forces as described in the main text, i.e. we let the single MT equilibrate at a fixed position, then let go and use the measured speed to deduce the resulting force. Fig C1-3 shows that this yields an excellent fit for the MT shapes and a very similar behavior of the calculated forces. The only meaningful discrepancy between the analytical and numerical results comes from the estimate of the y-component of the force. This is due to a specific way of clamping the MT in the nuclear envelope in Cytosim; note though that qualitatively the y-component of the force still has the same order of magnitude and sign in both approaches.

# References


1. Koke C, Kanesaki T, Grosshans J, Schwarz US, Dunlop CM. A computational model of nuclear self-organisation in syncytial embryos. J Theo Biol. 2014;359:92–100.

2. Mogilner A, Edelstein-Keshet L, Bent L, Spiros A. Mutual interactions, potentials, and individual distance in a social aggregation. J Math Biol. 2003;47(4):353–389.

3. Nédélec F, Foethke D. Collective Langevin dynamics of flexible cytoskeletal fibers. New J Phys. 2007;9(11):427.

4. Maly V, Maly I. Symmetry, stability, and reversibility properties of idealized confined microtubule cytoskeletons. Biophys J. 2010;99(9):2831–2840.

5. Maly IV. Equilibria of idealized confined astral microtubules and coupled spindle poles. PloS one. 2012;7(6):e38921.

6. Landau LD, Lifshitz EM. Theory of Elasticity, Vol. 7. Course of Theoretical Physics. 1986;3:109.
